# Intertwining social, affective, and digital dynamics: a masspersonal communication model to analyze home language maintenance

**DOI:** 10.3389/fpsyg.2025.1639079

**Published:** 2025-07-04

**Authors:** Jun-Yi Chen, Ting-Ting Liu

**Affiliations:** ^1^Fuzhou University of International Studies and Trade, Fuzhou, China; ^2^Chengdu University of Technology, Chengdu, China

**Keywords:** digital communication, family language policy, heritage language maintenance, home language development, masspersonal communication, multilingualism

## Abstract

In multilingual families, sustaining home languages is increasingly challenged by digitalization and evolving communication patterns. This study proposes a new analytical model to analyze how home language development and maintenance are shaped by three overlapping communication contexts: interpersonal, mass, and masspersonal communication. Grounded in the masspersonal communication model (MPCM) proposed by O’Sullivan and Carr, the model highlights how emotional attachment, interactive routines, and cognitive perceptions operate across these contexts. Interpersonal communication fosters intimacy and habitual language use; mass communication amplifies access to linguistic resources and influences parental ideologies through media exposure; and masspersonal communication, blending public and private dimensions, enables performative, collaborative, and feedback-driven practices that strengthen language identity and emotional ties. Practical recommendations for policy, education, and family language practices are outlined, emphasizing integrative approaches to leverage these intersecting forces. This study addresses a key theoretical gap by offering a model that captures how digitally mediated environments reshape language practices at home. Future research is encouraged to empirically validate the model and to trace affective and cognitive dynamics in home language socialization. By revealing the complex interplay between digital technologies, social interactions, and linguistic identities, this study advances conceptual understanding of home language maintenance in an interconnected, multilingual world.

## 1 Introduction

In an increasingly interconnected world, the maintenance of home languages within multilingual families has emerged as a critical area of inquiry. The capacity of families to transmit heritage languages across generations hinges not only on linguistic practices but also on broader social, technological, and ideological dynamics. Within this context, the role of family language policy (FLP) has been widely recognized as a central mechanism in sustaining linguistic diversity at home. FLP encompasses the deliberate strategies employed by parents to shape their children’s linguistic environment, strategies that are increasingly influenced by globalization, migration, and advances in digital communication technologies (Bose et al., 2024; Et-Bozkurt and Yağmur, 2022).

Notably, language ideologies strongly link heritage language retention to the affirmation of cultural identity and the pursuit of socioeconomic opportunities. For example, research on Latina and Mexican American mothers illustrates how language maintenance is closely tied to preserving cultural heritage while countering prevalent monolingual ideologies (Macias, 2023). However, the success of language transmission efforts depends not solely on parental choices; children’s active participation in linguistic interactions—commonly referred to as child agency—also plays a crucial role (Zhan, 2023; Said and Zhu, 2019). By adjusting their language use according to interlocutors and context, children become co-constructors of their linguistic environment (Quay, 2008).

The emergence of digital communication platforms has further reshaped the landscape of family language practices. With tools such as Skype, WhatsApp, and other digital media now integral to transnational family interactions, new forms of “networked family language policy” have surfaced (Bose et al., 2024; Lexander and Androutsopoulos, 2023). These technologies not only facilitate connections across geographic distances but also enhance the emotional bonds crucial for heritage language retention. However, while language maintenance may not always be the primary motivation for digital communication, its incidental benefits for sustaining linguistic ties are evident.

Moreover, digital media have expanded the resources available for language learning. Educational applications, online videos, and interactive games offer multilingual families greater access to heritage language materials (Obojska and Vaiouli, 2023). Parents generally perceive these tools as beneficial, though concerns about content appropriateness remain (Romanowski, 2025). Importantly, digital environments foster complex multilingual practices, such as translanguaging and code-switching, which allow families to navigate different linguistic repertoires for diverse communicative purposes (Karpava et al., 2019; Choi, 2019). These practices are instrumental in reinforcing language competence and cultural continuity.

Against this backdrop, the traditional divide between interpersonal and mass communication is becoming increasingly blurred. The masspersonal communication model conceptualizes this shift, highlighting how digital platforms blend personalized and public forms of communication (O'Sullivan and Carr, 2018). As families integrate digital tools into their daily interactions, new dynamics of “being together” emerge, reshaping family bonds and identity constructions (Bărbuță et al., 2023).

Despite these advances, challenges remain. The dynamic nature of FLP, influenced by social change and technological developments, complicates language maintenance efforts (Pittman and Glimois, 2024; Bose et al., 2023). Factors such as parental resources, generational differences, and the advice of educational professionals further intersect with the intricacies of maintaining a home language (Hollebeke et al., 2023). Nevertheless, multilingualism within families has been associated with numerous benefits, from positive social outcomes to deeper self-understanding and enriched interpersonal relationships (Burck, 2004).

Given the evolving complexities of home language development and maintenance, this article proposes a new analytical model based on the Masspersonal Communication framework. This model is designed to interpret how multilingual families navigate language practices across three interrelated communicative contexts: interpersonal, masspersonal, and mass communication. By doing so, it offers a conceptual lens to analyze how contemporary families negotiate language transmission in an increasingly digitized and networked society. The model aims to support empirical interpretation and enrich theoretical discussions, especially in examining how affective and cognitive dimensions shape language behavior in the home.

This article is conceived as a conceptual study, aiming to propose a theoretical model that integrates emotional, interactive, and cognitive dimensions of home language maintenance through the lens of masspersonal communication. The model is developed based on a synthesis of prior scholarship in sociolinguistics, communication studies, and heritage language research. Although no new empirical data are presented, the framework serves as an analytical foundation for future investigations, offering conceptual clarity in a field marked by increasing complexity and contextual variability.

At this stage of home language research, such a theoretical intervention is both appropriate and necessary. Current scholarship often treats interpersonal, mass-mediated, and hybrid communicative practices as disparate or competing domains, resulting in fragmented analytical approaches. With the growing prevalence of digitally mediated family interactions, emotionally charged language performances, and community-based language activism, there is an urgent need for a model that reflects the interconnectedness and dynamism of contemporary language practices.

The proposed model addresses this need by offering a flexible conceptual scaffold capable of capturing how various communicative contexts contribute to language maintenance. It also facilitates the exploration of how emotional attachments, interactive routines, and metalinguistic awareness are co-constructed across private, public, and semi-public domains. By bridging gaps between interpersonal discourse, media influence, and hybrid communicative performances, the model contributes to a more comprehensive understanding of language practice in multilingual families and sets the stage for future empirical inquiry.

## 2 Theoretical framework

The masspersonal communication model, developed by O'Sullivan and Carr (2018), offers a nuanced perspective on the evolving interplay between mass communication and interpersonal communication. The term masspersonal communication refers to a hybrid form of communication that combines the public reach of mass media with the interpersonal specificity of personalized messaging. The concept was developed to capture new communicative dynamics emerging from digital platforms, such as when users post intimate content to large audiences (e.g., a heartfelt family update on Facebook or a video of a child speaking a heritage language on Instagram). This mode of communication is simultaneously broad and intimate, allowing for highly personalized expression to occur in public or semi-public digital spaces. In this study, the term is applied to describe communicative practices that sit between private family talk and traditional mass media broadcasting, such as sharing a child’s heritage language use through social media, or participating in language challenges that circulate within community networks online. While the terminology stems from communication theory, it is used here as a pragmatic tool to describe everyday language activities that many multilingual families already engage in.

This study adapts the MPCM specifically to examine how multilingual families negotiate home language maintenance in the context of digital communication, positioning the model as an analytical tool for exploring affective, interactive, and cognitive dimensions of language practice. Rather than treating these communication modes as distinct, the model integrates two dimensions—perceived message accessibility and message personalization—allowing communication acts to be positioned along a continuum independent of traditional channel-based distinctions. Through this framework, interactions are no longer rigidly classified by medium but are understood through the degree of personalization and potential audience reach.

At its core, MPCM addresses the fluidity of communication in contemporary digital environments. The intersection between mass and interpersonal communication becomes particularly evident in settings where individuals simultaneously engage broad audiences while maintaining personalized interactions. For instance, social media platforms embody this hybrid communication form, enabling users to craft messages that, although publicly accessible, maintain a sense of intimacy and personal relevance (French and Bazarova, 2017; Zell and Moeller, 2018). This intersection challenges conventional boundaries, underscoring the importance of understanding not just how messages are transmitted but also how they are perceived in terms of accessibility and personal relevance.

The applicability of MPCM extends beyond social contexts into educational settings, especially in language learning and maintenance practices. Traditional face-to-face (F2F) communication, long regarded as essential for language acquisition due to its immediacy and rich feedback loops (Chew and Ng, 2021), now coexists with computer-mediated communication (CMC). CMC platforms offer synchronous and asynchronous opportunities for language interaction, expanding the potential for learners to engage with diverse interlocutors beyond geographical constraints (Hung et al., 2022). The MPCM framework effectively captures this evolution by situating these communication practices along its dual-axis model, revealing how digital interactions can replicate or diverge from traditional interpersonal dynamics.

Moreover, MPCM’s focus on anticipated audience response—a key element in masspersonal interactions—enhances its relevance for language education (French and Bazarova, 2017). Learners are increasingly aware of and motivated by the potential for feedback in digital environments, shaping how they engage with language learning tasks. Social media platforms, for instance, not only facilitate language practice but also create spaces where learners anticipate validation or correction from peers and broader audiences, thus reinforcing their communicative competence.

Within educational pragmatics, communicative competence is reinterpreted through the lens of MPCM, where effectiveness in interaction hinges on strategic language use and the management of relational dynamics (Mara and Mara, 2011). The personalization dimension of MPCM aligns with the need for learners to tailor their language practices to diverse audiences, while the accessibility dimension reflects the broad dissemination potential inherent in digital media.

When adapted to the analysis of home language maintenance, MPCM provides a conceptual scaffold for investigating how multilingual families navigate complex digital and social landscapes. Its dual emphasis on personalization and public accessibility allows researchers to trace how family members use language to construct emotional connections, establish routines, and negotiate cultural identity within and beyond the household. This theoretical adaptation thus bridges macro-level media environments and micro-level family interactions, offering a holistic lens through which to interpret language socialization in a digitized world.

The masspersonal communication model, originally developed to address the convergence of mass and interpersonal communication in digital environments, emphasizes two primary dimensions: publicness and personalization. In its initial formulation, MPCM accounts for how digital platforms enable highly personalized messages to reach large audiences, thereby blurring traditional distinctions between public broadcasting and private interaction (O'Sullivan and Carr, 2018).

To adapt this model for the context of home language maintenance, the current study reconfigures its focus in three key ways. First, the model’s core dimensions are retained—publicness and personalization—but reinterpreted through the lens of language-related social practice, specifically how families strategically use communication modes to support heritage language continuity. Second, building upon the MPCM’s structural insights, three analytical domains—emotional dynamics, interactive practices, and cognitive meanings—are introduced to capture the multi-layered functions of communication in the home. These domains reflect not only the technological affordances of communication platforms but also the sociolinguistic intentions, cultural identities, and pedagogical roles embedded in everyday family discourse. Third, the model is extended by mapping three communication contexts—interpersonal, mass, and masspersonal—onto a continuum of language practices, each characterized by different configurations of affective engagement, routine behavior, and reflective meaning-making. This integration allows the MPCM to move beyond its original concern with digital behavior and become a heuristic tool for analyzing how heritage language ideologies are performed, negotiated, and transmitted across hybrid communicative environments.

Through these steps, the adapted model serves as both a conceptual bridge and an analytical framework, enabling researchers to systematically explore how families mobilize different communicative resources—ranging from private talk to public performance—to sustain linguistic and cultural continuity in increasingly mediatized societies.

## 3 Communication contexts shaping home language practices

Building on the masspersonal communication model, this section delineates three interrelated communication contexts—interpersonal, mass, and masspersonal—each shaping home language practices across emotional, interactive, and cognitive dimensions.

### 3.1 Interpersonal communication context

This section explores interpersonal communication as a primary context shaping home language practices through three interrelated dimensions: emotional, interactive, and cognitive. These dimensions are chosen to reflect the holistic nature of family communication, which simultaneously involves affective bonding, habitual language routines, and intergenerational meaning-making. Emotional dynamics refer to how language use fosters intimacy and emotional expression; interactional practices capture the routinized and agentive aspects of language use in daily life; and cognitive meaning encompasses the roles, responsibilities, and values attached to language within family structures. These dimensions together allow for a multi-faceted understanding of how interpersonal settings support heritage language maintenance.

#### 3.1.1 Emotional dynamics

The use of the home language (HL) within families plays a crucial role in shaping emotional bonds and fostering intimacy. Children’s emotional preferences for language are closely related to their proficiency in the heritage language, the linguistic practices of their parents and siblings, and their attitudes toward both the HL and the dominant institutional language (Dekeyser and Agirdag, 2021). When families maintain the HL, they not only preserve linguistic diversity but also reinforce emotional closeness.

Parent–child communication about emotions is a particularly important site where language and emotional development intersect. Studies have shown that discussions of emotional experiences between parents and children significantly contribute to children’s emotional competence (Wang, 2020). The language used during these conversations matters; heritage languages can provide a deeper emotional resonance, enhancing the quality of emotional exchanges (Chen et al., 2012). Fathers, often assumed to be less expressive, have also been found to play a vital role in such interactions, challenging stereotypes and underscoring the emotional importance of paternal communication in HL (Chen et al., 2012).

Children’s ability to understand and manage emotions is closely linked to their language skills, as the development of emotional literacy relies on the ability to label, describe, and reflect on emotional states—a process inherently shaped by language (Sevinç and Mirvahedi, 2023). Family language policies, therefore, not only reflect language ideologies but may also function as psychological coping mechanisms, especially in migrant families where maintaining the HL can serve to stabilize emotional identities amid sociocultural transitions (Tannenbaum, 2012).

Furthermore, children’s ability to understand and manage emotions is linked to their language skills. Parental use of mental-state language—talking about feelings, thoughts, and desires—often occurs in the HL and has been shown to support children’s emotional understanding and regulatory abilities (McQuaid et al., 2008; Aguert, 2022). In transnational families, emotional expressions are increasingly multimodal, combining HLs with symbols like emojis or mixing languages, which contributes to maintaining emotional closeness even across distances (Curdt-Christiansen and Iwaniec, 2023).

Importantly, parental language practices at home are predictive of children’s life satisfaction. Children who use the HL in daily interactions tend to report higher levels of well-being, suggesting that the emotional value of HL use extends beyond immediate family ties to broader aspects of psychological health (Humeau et al., 2025).

#### 3.1.2 Interactional practices

Beyond emotional connections, the formation of language routines within families is fundamental to sustaining HL use. Routine interactions—such as shared meals, bedtime stories, and household tasks—serve as predictable, recurring contexts for embedding language practice, providing opportunities for language reinforcement without explicit instruction (Lanza, 2007).

Family Language Policy—the explicit or implicit planning of language use at home—serves as a critical mechanism for embedding such routines (Pittman and Glimois, 2024; Schwartz, 2008). Regular and patterned use of the HL during routine activities creates stable opportunities for language practice and reinforces linguistic patterns (Quay, 2008).

Children are increasingly recognized as active participants in these interactions. Their attitudes, preferences, and contributions co-construct the linguistic environment, shaping how and when the HL is used (De Houwer, 2007). Children may respond creatively to adult language input, initiate code-switching, or even influence parental behavior—highlighting the bidirectional nature of family language socialization.

This perspective aligns with research on intentional and adaptable FLPs, emphasizing the need for families to adjust their language strategies as circumstances change, including embracing digital media for networked family interactions (Bose et al., 2024; Torsh, 2025).

Structured interventions such as Routine Language Intervention have shown that incorporating rich and responsive language into daily family routines—by guiding parents to engage in meaningful, consistent interactions—can be just as vital as formal educational settings in promoting children’s heritage language development, underscoring the importance of everyday language use in fostering vocabulary acquisition and communicative competence through naturally embedded routines within the home environment (Masek et al., 2024; Domeniconi and Gràcia, 2018).

#### 3.1.3 Cognitive meaning

The division of language roles within families often reflects broader intergenerational responsibilities for language transmission. Grandparents, for instance, play a significant part in heritage language maintenance, particularly in contexts where children spend time with them during formative years (Kutsaeva, 2024; Ruby, 2012). In many cases, grandparents are not only language transmitters but also custodians of cultural values and traditions.

Parents, meanwhile, often act as “medium translators,” bridging linguistic gaps between older and younger generations, sometimes facilitating a shift toward dominant languages (Wang and Curdt-Christiansen, 2021). In multilingual households, this mediation can inadvertently result in language shift if not carefully managed, especially when dominant societal pressures and educational systems discourage HL use (Blacher and Brehmer, 2024).

Children also bear a degree of intergenerational responsibility. Studies from Flanders show that second-generation family members often carry the dual burden of maintaining their HL while integrating into the majority language community (Verhaeghe et al., 2022). Effective FLPs that foster positive emotional ties with HL and involve all generations can mitigate the risks of language attrition (Lubis, 2024).

Altogether, interpersonal communication contexts—emotional bonding, routine practices, and meaning-making roles—form a dynamic infrastructure for HL maintenance. These interpersonal processes not only promote language sustainability but also fortify familial identity and emotional resilience.

### 3.2 Mass communication context

Mass communication operates on a broader social scale, influencing family language practices through public representations, accessible resources, and shifting language ideologies. Media platforms play a pivotal role in shaping collective attitudes toward language status and usage, offering both opportunities and challenges for heritage language preservation. This section explores the emotional, interactional, and cognitive impacts of mass communication on home language development.

#### 3.2.1 Emotional dynamics

The portrayal of minority languages in media significantly impacts emotional dynamics by fostering language pride and reinforcing cultural identity. Media visibility of minority languages can either elevate or diminish speakers’ sense of belonging. In Lesotho, for instance, the underrepresentation of minority languages such as SiPhuthi and isiXhosa on national radio—where Sesotho and English dominate—has contributed to the marginalization of these language communities, dampening their linguistic pride (Nkhi et al., 2025). In contrast, platforms like Alian FM 96.3 in Taiwan actively incorporate indigenous languages, helping indigenous youth develop a stronger sense of cultural pride (Lin, 2023).

Beyond representation, media also influences emotional responses by shaping perceptions of language status. Community radio stations in South Africa that broadcast in indigenous languages enhance listeners’ cultural affinity and emotional bonds, fostering greater inclusion and participation (Onyenankeya and Salawu, 2023). Similarly, media initiatives in Zimbabwe, such as code-switching practices on Star FM’s Breakfast Club Show, allow for linguistic expressiveness, although access remains uneven across dialects (Mukenge, 2025). In Sweden, the inclusion of minority languages and sign language in public children’s programming showcases how thoughtful media policies can promote inclusivity (De Ridder, 2024).

Digital technology further amplifies the potential for emotional engagement through language. Applications like the “Koryak tuyu” app in Kamchatka provide endangered language communities with tools for revitalization, creating emotional connections to cultural heritage (Fayzrakhmanova, 2024). Moreover, comparative studies show that Russophone families across Estonia, Germany, and Sweden report that digital technologies have intensified emotional ties by facilitating intergenerational communication and supporting heritage language practices among youth, particularly through online interaction with extended family members (Ringblom et al., 2024).

#### 3.2.2 Interactional practices

The role of media in facilitating language learning is multifaceted, operating as both a resource and a risk. High-quality educational media can support bilingual language development by introducing children to second language cultures, enhancing vocabulary acquisition, and fostering broader cultural competencies (Saleem and Khan, 2024; Patel et al., 2025). When children engage with educational television content—especially programs featuring captions—they experience incidental learning, which can substantially boost vocabulary and listening skills (Paradita et al., 2025; Muñoz et al., 2023; Peters, 2019).

However, not all media exposure is beneficial. Background television and excessive screen time can negatively impact language development by displacing traditional literacy practices like shared reading and reducing opportunities for social interaction (Schlesinger et al., 2019; Linebarger and Vaala, 2010; Dore et al., 2020). To mitigate these risks, co-viewing—watching media with adult guidance—has been shown to enhance learning outcomes by replicating the dynamics of live, interactive communication (Linebarger and Vaala, 2010).

Digital resources also play an expanding role in heritage language revitalization. Community-driven initiatives such as the Kaytetye Indigemoji app demonstrate the effectiveness of integrating local knowledge into language learning tools (Lea et al., 2025). Additionally, projects like SiDHELA, which develop localized digital archives in collaboration with indigenous communities, provide sustainable platforms for language documentation and revitalization (Karthick Narayanan and Takhellambam, 2022). Educational platforms leveraging cultural heritage open data further enrich the learning environment by offering multimedia materials that blend language and culture (Ferrara et al., 2014).

#### 3.2.3 Cognitive meaning

Media exerts profound influence on parental language ideologies, reshaping family language policies and children’s language outcomes. Parental beliefs about language are closely tied to their linguistic identities and experiences, shaping the goals and strategies they set for their children’s multilingual development (King et al., 2008; Ellis and Sims, 2022).

However, there can be a divergence between parents’ intentions and children’s actual language use, often due to external influences like schooling systems (Maseko and Mutasa, 2018; Cangelosi et al., 2024). For example, children may be encouraged to use the heritage language at home, while experiencing institutional pressure to speak only the dominant language in school settings. Such mismatches are well-documented in research on family-school language conflict (Wesely, 2018; Pagé and Noels, 2024), where the child’s social environment leads to a gradual shift in linguistic preference, even in households with strong HL ideologies. The result is often frustration among parents and emotional disengagement in children, creating strain on intergenerational language continuity.

Media not only informs but also transforms language beliefs. It disseminates cultural values and societal norms, subtly influencing attitudes toward heritage and dominant languages (Arias, 2019; Stacks et al., 2015). Through this influence, media can shift identity alignments, as increased exposure to dominant-language media correlates with a stronger identification with dominant cultural groups (Clément et al., 2005). Moreover, media contributes to the construction of societal perceptions, shaping collective beliefs about social issues (Shehata, 2021).

In the realm of education, heritage language learning has embraced translingual and critical pedagogy approaches to counterbalance these media-driven influences. Collaborative translation practices, where bilingual students work together to interpret texts and share meanings, enhance bilingual reading skills and metalinguistic awareness (Puzio et al., 2013; Cano and Ruiz, 2020). Strategies like collaborative translation and project-based learning help students navigate complex linguistic identities and resist dominant language pressures (Wu and Leung, 2022; Cushing-Leubner, 2019). Similarly, project-based learning (PBL) has been shown to strengthen bilingual students’ literacy and engagement, particularly when it incorporates family participation and culturally relevant materials (Beckett, 2024; Alvarez, 2023). Community-based experiential learning further anchors heritage language education in local realities, supporting both learners and communities (Guerrero-Rodriguez et al., 2021; Aravossitas, 2023). These practices not only develop language skills but also reinforce identity and agency.

In sum, the cognitive domain of mass communication is shaped by the complex negotiation of ideologies, expectations, and educational influences. By highlighting examples of parental-child mismatches and culturally responsive interventions, this section underscores how media-related cognitive processes can both challenge and support heritage language maintenance.

### 3.3 Masspersonal communication context

Masspersonal communication occupies the intersection between interpersonal intimacy and mass-mediated outreach, offering unique spaces where language practices are performed, co-constructed, and reshaped through feedback mechanisms. This section analyzes how digital platforms facilitate emotional dynamics, interactive engagement, and cognitive meanings in the development and maintenance of home languages.

#### 3.3.1 Emotional dynamics

Social media has become an essential platform for performing and displaying family languages and identities. Family enterprises, for example, strategically leverage visual and textual representations to foster brand authenticity and deepen customer engagement online, illustrating how language practices are closely tied to digital self-presentation (Zanon et al., 2019). Similarly, the use of social media by indigenous communities for promoting languages like Punjabi and Setswana demonstrates its vital role in cultural preservation and community building (Minhas and Salawu, 2024). Even in families separated by distance, social media rituals and storytelling sustain bonds and reinforce family identity (Abel et al., 2021). These examples underscore the performative nature of language in digital communication, where expressions range from formal self-branding to emotional displays through emojis, enhancing nuanced interpersonal connections (Liu et al., 2024). Moreover, language in digital interactions is pragmatically structured; utterances are not mere exchanges but intentional performances that signal user intentions and community participation (Cui and Mao, 2017; Cui et al., 2017). In this view, masspersonal communication fosters a digital performativity where language is not only a medium of communication but also a form of cultural and emotional articulation.

#### 3.3.2 Interactive practices

Masspersonal contexts also foster interactive language practices through co-creation. In education, co-creation involves collaborative development of curricula and teaching practices between students and staff, enhancing inclusivity and learning outcomes (Omland et al., 2025; Shelton et al., 2023). This model mirrors heritage language initiatives, where community-driven learning connects language acquisition to sociocultural experiences (Montrul, 2023; Montrul and Polinsky, 2021). Heritage language learning thrives on community-based activities that intertwine cultural knowledge with language practice, demonstrating that identity and language are co-constructed through engagement (He, 2010; O'Connor, 2012). Furthermore, user-generated content in digital heritage projects highlights the importance of multicultural participation for effective language and cultural preservation (Rahaman and Tan, 2011). Addressing linguistic marginalization through multimedia initiatives has proven effective in revitalizing minority languages and fostering community identity (Palmer-Wackerly et al., 2014; Mendoza et al., 2024). Thus, in masspersonal environments, language is a collaborative enterprise, shaped by collective action and co-constructed meaning-making.

#### 3.3.3 Cognitive meanings

Language use in masspersonal communication contexts is also deeply shaped by feedback dynamics, influencing cognitive perceptions of language and identity. Studies show that immediate and delayed feedback significantly boost motivation and learning outcomes, especially when coupled with positive affective language (Qun, 2025; Nguyen et al., 2017; Cen and Zheng, 2024). Metalinguistic corrective feedback further enhances willingness to communicate, while self-feedback correlates strongly with learning motivation (Montazeri and Salimi, 2019; Gan et al., 2023). On social media, likes, comments, and shares act as immediate feedback mechanisms that reinforce heritage language practices and foster vibrant online communities (Baitalik, 2025). Meanwhile, parental attitudes play a crucial role in maintaining heritage languages, with social environments and community support acting as reinforcing agents (Listiana and Armielia, 2024; Weekly, 2020). Affective factors, such as language anxiety and positive emotional experiences, deeply influence language identity and learning persistence (MacIntyre and Gregersen, 2012). Furthermore, heritage language maintenance is closely tied to identity preservation, where cultural and linguistic experiences reinforce a dual or hybrid cultural self (Brehmer, 2021; López-Beltrán and Carlson, 2020; Lee, 2010). Feedback in digital spaces thus not only motivates linguistic engagement but also shapes cognitive structures and cultural identification.

In sum, masspersonal communication spaces provide a rich environment for the performative display, collaborative construction, and feedback-driven shaping of language practices. These dimensions work collectively to reinforce language use, cultural identity, and emotional connections in an increasingly digitalized and interconnected world.

## 4 Discussion

**Figure 1:**
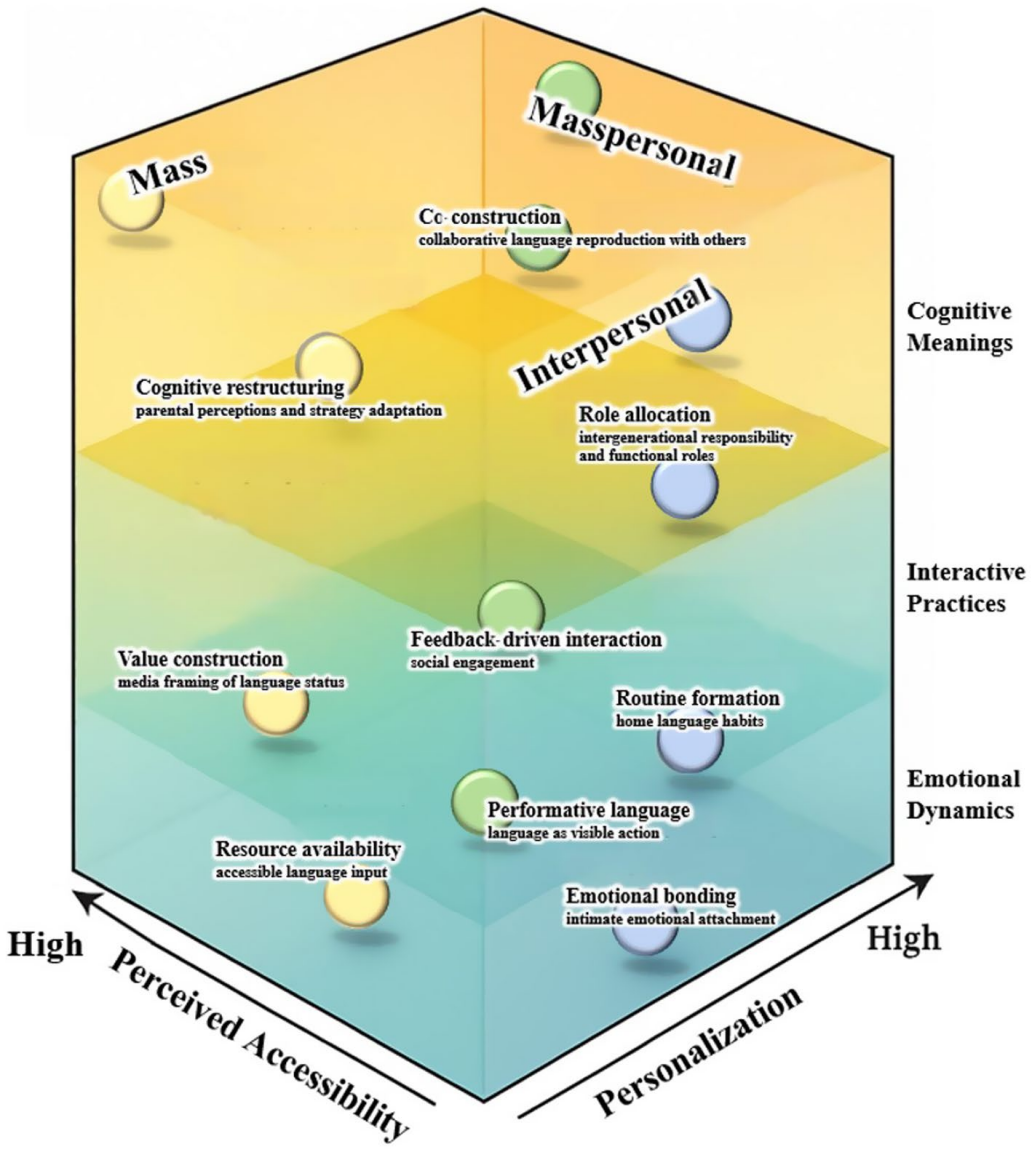
A masspersonal communication model. The vertical axis represents language practice dimensions, including emotional dynamics, interactive practices, and cognitive meanings. The horizontal axes reflect perceived accessibility (from low to high) and personalization (from low to high). Each communication context is associated with specific functional roles across these dimensions, visualized through spatial distribution.

To clarify the analytical scope of this study, this section reconsiders the three communication contexts—mass, interpersonal, and masspersonal—in light of their emotional, interactive, and cognitive contributions to home language maintenance. This multidimensional approach aligns with the study’s overall aim of developing a masspersonal communication model that captures how families engage with language practices across personal, social, and media environments. To support this analysis, Table 1 and Figure 1 are introduced below to illustrate the relationships between communication types, heritage language practices, and their operational dimensions.

**Table 1:** Communication types and corresponding language practices.

Communication type	Publicness	Personalization	Corresponding language practices
Interpersonal communication	Low	High	Parent–child conversations, daily home use of the heritage language, transmitting songs via grandparents
Mass communication	High	Low	Watching heritage language programs as a family, using official teaching materials, audiobooks
Masspersonal communication	High	High	Parents sharing videos of children speaking the heritage language on social media, participating in language challenges, public sharing of voice messages

Masspersonal communication refers to communicative acts that are simultaneously personalized and publicly accessible—such as publicly sharing intimate family language moments online. This hybrid category bridges private and mass-oriented practices. Publicness refers to the extent to which communication is accessible to a broad audience, while personalization refers to the degree of tailoring communication to individual recipients. Examples illustrate typical heritage language practices associated with each communication type.

Table 1 outlines how mass, interpersonal, and masspersonal communication differ in terms of perceived accessibility and personalization, linking these types to corresponding language practices observed in family and community contexts. These distinctions provide the foundation for a deeper analysis of the underlying mechanisms that sustain heritage language use across varying communicative landscapes.

Figure 1 visualizes the interplay among the three communication types and their contributions to three key functional domains—emotional dynamics, interactive practices, and cognitive meanings. The spatial layout illustrates how communicative contexts distribute along the axes of perceived accessibility (mass vs. interpersonal) and personalization, with masspersonal communication occupying a hybridized and fluid position between the two.

Together, these representations provide a cohesive framework that operationalizes how emotional bonding, home language routines, and meaning negotiation emerge from different communicative environments.

The interaction among these three contexts—mass communication, interpersonal communication, and masspersonal communication—reveals a dynamic and mutually reinforcing system of heritage language practices. These contexts do not function in isolation; they intersect to shape language ideologies, emotional investment, and identity construction. For instance, the emotional power of media representation may generate heritage language pride, but such pride is further sustained by interpersonal practices such as parental language input and masspersonal expressions like sharing children’s language performances on social platforms.

This interdependence illustrates the necessity of adopting a holistic and integrated view, where mass dissemination, intimate engagement, and hybridized communication reinforce each other in the construction of resilient language environments. The proposed model not only maps these relationships, but also provides a structure for identifying where breakdowns or strengths in transmission might occur.

In terms of operational guidance for empirical research, each communication context can be studied through the three functional dimensions as integrated categories of observation and analysis. Emotional dynamics may be explored by examining affective responses to language practices, such as expressions of pride, intimacy, or nostalgia toward the heritage language. Interactive practices can be traced through patterns of turn-taking, the frequency and nature of participation, or habits related to content creation and sharing. Meanwhile, cognitive meanings may be assessed through narrative framing, instances of metalinguistic reflection, or how language users articulate strategic decisions regarding language use. These dimensions, taken together, offer a comprehensive and adaptable framework for designing empirical studies that capture the complexity and variability of multilingual family communication. These dimensions offer coding categories for qualitative data analysis and variables for quantitative studies, enabling the model to serve as both an interpretive and predictive tool.

Practical applications can also be drawn from this framework by translating its insights into actionable strategies across educational, familial, and policy domains. For example, educators may integrate heritage language instruction with project-based learning that incorporates masspersonal communication tools, such as voice blogs, video storytelling, or classroom-based digital exhibitions. These activities not only reinforce language production but also engage students in performative, audience-centered practices that reflect real-world communication. Parents can apply similar strategies by capturing and sharing children’s heritage language use within closed digital communities or with extended family members through messaging platforms, thereby embedding linguistic routines into emotionally resonant, semi-public rituals. At the policy level, institutions can support such practices by promoting media literacy initiatives that include guidance on safe, inclusive, and culturally responsive digital participation. Toolkits and awareness campaigns may help caregivers better navigate the affordances of social technologies while fostering positive linguistic identities. Crucially, such recommendations must remain sensitive to varying levels of digital access and cultural comfort with public visibility, ensuring that proposed solutions are flexible and context-appropriate.

While the proposed model offers an integrated framework for understanding the interplay between communication contexts and home language practices, several limitations warrant careful acknowledgment. First, although the framework captures a wide range of communicative scenarios, it does not fully account for structural and sociopolitical constraints, such as unequal access to digital technologies, platform-specific affordances, or language hierarchies embedded in educational and policy systems. These structural factors significantly shape how families engage—or are constrained from engaging—with the communication modes outlined.

Second, the diverse sociolinguistic ecologies in which multilingual families operate may involve substantial variation in digital literacy, cultural norms, and media use practices, all of which influence the applicability and salience of each communication context. For example, families with limited digital resources may find masspersonal communication less accessible or meaningful, whereas others may rely heavily on such hybrid forms to sustain language connections.

Third, the model does not currently address the full spectrum of interactional dynamics, such as peer-group influence, intergenerational tensions, or the role of institutional mediation (e.g., schools or religious organizations). Moreover, the dynamic and fluid nature of communication may lead to overlaps that blur the categorical distinctions proposed in the model—particularly in hybrid interactions such as livestreamed parent–child dialogues or family messaging groups involving extended kin networks.

These limitations do not undermine the utility of the framework but rather underscore the need to view it as a heuristic lens, adaptable and sensitive to context-specific modifications. Future empirical work could refine the model by incorporating these dimensions and testing its relevance across different sociocultural settings. As such, the current model offers a necessary conceptual intervention while inviting ongoing dialogue and localized adaptation in future research.

To strengthen the model’s empirical utility, future research may explore three complementary directions. First, qualitative studies such as in-depth interviews or ethnographic observations can investigate how multilingual families navigate communication contexts in everyday life, revealing nuanced affective and interactive patterns. Second, quantitative surveys could examine the relationships between emotional bonding, media use patterns, and heritage language attitudes, providing a broader understanding of contextual influences. Third, longitudinal mixed-methods designs may track changes in emotional affiliation, cognitive strategies, and communication routines across developmental stages or life transitions. These approaches offer an integrated roadmap for scholars seeking to operationalize and test the model, particularly across diverse sociocultural and technological environments.

In conclusion, the proposed masspersonal communication model offers an integrated analytical lens for understanding home language practices. It articulates the intersection of media, personal engagement, and hybrid communication in shaping emotional, interactive, and cognitive dimensions of heritage language maintenance. By visualizing these interrelations, the model supports both theoretical synthesis, paving the way for future cross-contextual and longitudinal studies of multilingual development.

## Data Availability

The original contributions presented in the study are included in the article/supplementary material, further inquiries can be directed to the corresponding author/s.
